# Simple technique for fabrication of shielding blocks for total body irradiation at extended treatment distances

**DOI:** 10.4103/0971-6203.56084

**Published:** 2009

**Authors:** R. Ravichandran, J. P. Binukumar, C. A. Davis, A. M. Zahid, B. Rajan

**Affiliations:** Medical Physics Unit, National Oncology Center, Royal Hospital, Muscat, Sultanate of Oman; 1 Department of Radiation Oncology, National Oncology Center, Royal Hospital, Muscat, Sultanate of Oman

**Keywords:** Magna field radiotherapy, shielding in TBI, total body irradiation

## Abstract

Techniques are being standardized in our department for total body irradiation (TBI) with six MV photons in linear accelerator for preconditioning to bone marrow transplantation (BMT). Individualized shields with low melting point alloy are to be fabricated for shielding critical organs such as lungs, kidneys etc. A method to mount diminished dimension of shields in a tray at 3.75m is designed in the department for a teletreatment distance of four meters with magna field with A simulator image taken with the patient's midplane (MP) at one meter distance is used to mark the dimensions of lung, scaled down by a factor of 3.75/4.0. These lung dimensions are reprinted from the digital simulator image for making the shield. The methodology of the technique using digitized minification in radiography is the first of its kind to be used for shield cutting in magna field radiotherapy.

## Introduction

Radiotherapy with large fields like Mantle in the treatment of Hodgkin's Disease or total body irradiation (TBI) in the treatment of hematological malignancies necessitates the need for shielding critical organs such as lungs, kidneys etc. Individualized shields with low melting point alloys are normally fabricated in the mould room using styrofoam cutting system. To simulate the conditions of treatment, teleradiography needs to be carried out with the same divergence as the treatment beam.[1,2] The difficulty in reproducing radiographs at nonstandard treatment distances could be overcome using a tray-shift technique.[3] Ravikumar and Ravichandran[4] investigated the inaccuracy in the shielded volumes in such a technique. The need for standardizing dosimetry for TBI in a newly started radiotherapy center has been explained in the reports.[5,6] The technique for developing shields for extended treatment distance (four meter) becomes necessary for these treatments. There is no standard method available for fabrication of such shields. At the treatment distance of four meters the photon beam is almost parallel with an insignificant divergence. However, the shields fabricated should be accurate enough to exactly cover the region of interest. Any error in the shield may give rise to excess dose in the organ to be shielded or sometimes lead to unnecessary shielding of target volumes. This paper outlines a simple method to fabricate shields of correct dimensions to shield both lungs in TBI treatment.

## Materials and Methods

### Patient Treatment

**Figure 1:**
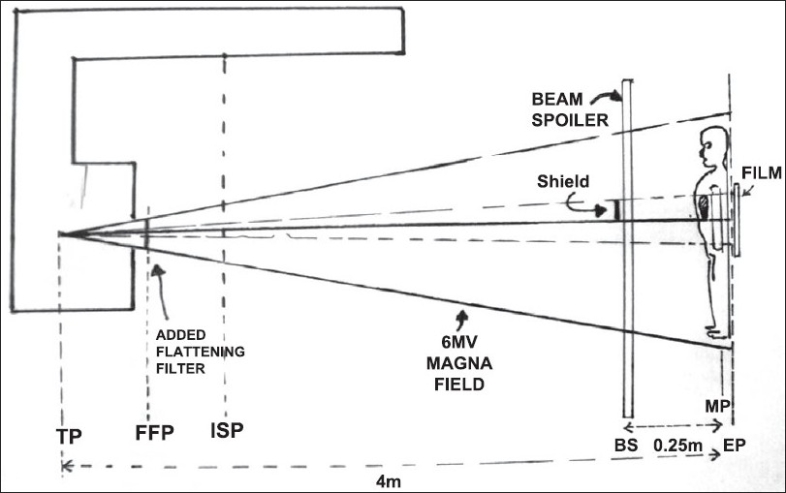
Geometry of TBI Treatment Showing Position of Shield

The TBI is planned with the patient immobilized in the treatment position at a target to mid-plane distance of four meters with a six MV photon beam from clinac 600 CD linac (Varian AG, USA) with Gantry angulation 270° and collimator angulation 45° to achieve diamond shaped photon field. The patient is positioned on a stand-alone treatment couch with motorized vertical movements at the extended treatment distance. The patient lies on his side, left as well as right, to facilitate beam entry in anteroposterior (AP) and posteroanterior (PA) directions. The central axis of the magna-field (40 cm × 40 cm field opening at one meter isocenter plane) passes through the umbilicus (reference point) of the patient. A beam spoiler (BS) of dimension 2m × 0.7m × 0.015m[6] is positioned 25 cm in front of the patient, at a distance of 3.75 m from the target of linac. The geometry of the planned treatment is shown in Figure 1.

### Position of Shield

The tray on the beam spoiler to hold both lung shields is kept at a distance of 3.75 meters from the target and at 25 cm in front of the patient's midplane (MP) [Figure 1]. The factor of divergence (4.0m/3.75m =1.066) is about seven per cent and is accounted for, while the dimensions of the shield are calculated.

### Drawing Dimensions of Shield

**Figure 2:**
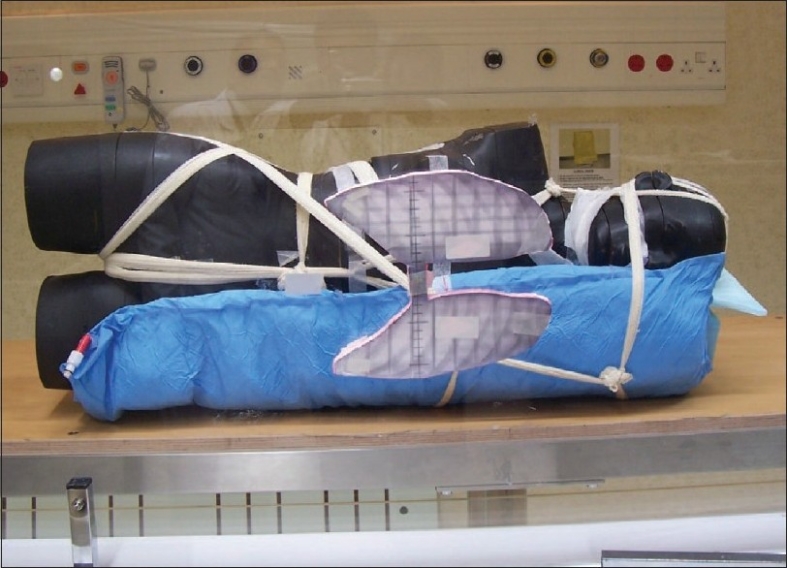
Position of Styrofoam Shield Template on Beam Spoiler in Verification Radiography

An Acuity simulator (M/s Varian AG, USA) has provision for acquisition of radiograph digitally using electronic imaging system. The volume of lung to be shielded is delineated on the radiograph acquired in the simulator with the patient's midplane positioned at an isocenter distance of one meter using Varis-Vision (Varian Ag, USA) tools with the required magnification factor. The patient's MP is positioned at four meters and the shield is placed at the position of beam spoiler (BS) plane [Figure 2]. The required minification factor on the digital image of the lungs is 3.75/4.0 is equal to 0.9375. The shields are fabricated for the minified size so as to provide correct shielding dimensions on the patient's MP.

### Validation of Accuracy of Shield

The accuracy of the shields is checked with the same print out by outlining the delineated lung with a thick lead wire of the shield fixed on the special tray mounted on the beam spoiler. A humanoid phantom (Alderson-Rando phantom) placed in the treatment position simulating the patient is radiographed with the developed shield in position. A film (Kodak V X-Omat) loaded in a film cassette (Agfa Gavaert) kept at exit plane (EP) of the phantom, 5 cGy dose is delivered at the position of the cassette (75 MU) at 300 MU/min rep-rate.

## Results

**Figure 3:**
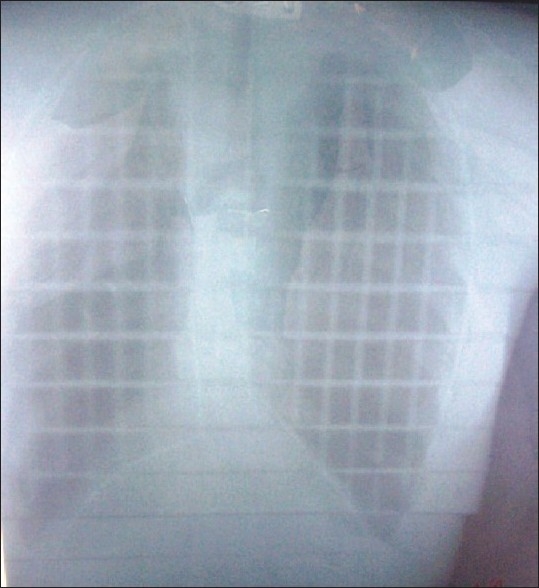
Verification Radiograph with Lead Wire Positioned around Periphery of Styrofoam Shield Template

The radiograph obtained is shown in Figure 3. It can be seen that the outline of the lead wire image covers exactly the lung region of interest and the method is acceptable for the treatment.

## Discussion

This paper has outlined a simple method, which can be implemented, in delivering TBI at extended distances. The methodology of the technique using digitized minification in radiography is the first of its kind to be used for shield cutting in magna field radiotherapy. The routinely used methods are use of either:

1. a stryrofoam cutting machine to cut the shield shape accounting for divergence or
2. empirically place approximate dimension of shield outline with respect to central axis and later correction of shapes based on the portal verification film patterns

As the true dimensions of internal organs remain the same irrespective of the distance at which the treatment is planned, the divergence should be accounted at any distance where the shield is placed. As this method is based on standard geometry, the adequacy of shielding is assured. The alignment of central axis of the beam and position of the shields are easier. The accuracy achievable in drawing the shield dimension is equivalent to the spatial accuracy achievable with the modern therapy simulator. This procedure can be implemented in any department having a digital simulator. The shielding can be verified at the first few cGy doses which can be carried out during treatment delivery in progress.

As we have used only diagnostic rare earth film with corresponding intensifying screen for megavoltage radiograph, the dose of 5 cGy to film was sufficient to give good quality radiograph. Since verification is possible before actually casting the shield with metal alloy, this method is acceptable in all departments. The earlier described tray-shift technique[3] is applicable mainly for short target-tray distances. This is difficult to apply at very large distances where the styrofoam cutting system cannot be useful. We recommend this method for TBI treatments.

The methodology of obtaining dimensions of shields for protecting specific organs has been outlined. We plan to use Lipowitz metal (brand name Cerroband shielding alloy, also known as low melting point alloy) for fabrication of shields. This alloy has density 9.4 g/cm,[3] consisting of bismuth 50%, lead 26.7%, tin 13.3% and cadmium 13.3% in composition. The measured half value thickness for six MV photon under broad beam conditions is 16 mm. Depending on the transmission requirements for including lung dose, shields of different thickness will be planned. This report will form the basis for obtaining clearance of our planned TBI treatment protocol in the department. Hence, we could not give a radiograph of actual patient undergoing treatment
